# Un équivalent de Galeazzi chez l'enfant: une entité clinique rare

**DOI:** 10.11604/pamj.2014.19.295.5318

**Published:** 2014-11-17

**Authors:** Mechchat Atif, Elmrini Abdelmajid

**Affiliations:** 1 Departement de Chirurgie Orthopédique et Traumatologie B4, CHU Hassan II, Fez, Maroc

**Keywords:** Galeazzi équivalent, enfant, avant bras, fracture, equivalent Galeazzi, child, forearm, fracture

## Image en medicine

Un garçon de 14 ans se présente aux urgences pour une impotence fonctionnelle douloureuse de l'avant bras droit à la suite d'une chute de sa bicyclette. L'examen clinique initial a montré un avant bras tuméfié avec une vive douleur au tiers moyen sans déficit vasculaire ou nerveux. Les diagnostics à évoquer sont une fracture du radius ou de l'ulna ou des deux os de l'avant bras. La radiographie de l'avant bras de face et de profil a montré une fracture de la diaphyse radiale avec une angulation antérieure, associée à un décollement épiphysaire stade II de Salter et Harris de l'extrémité distale de l'ulna. Le diagnostic final est une fracture équivalente à la fracture de galeazzi. Le mécanisme le plus probable est une chute sur la paume de la main avec une extension du poignet, et une pronation forcée de l'avant-bras avec un coude fléchi. Les forces sont transmises vers le poignet, produisant ainsi une fracture de la diaphyse radiale au niveau de sa courbure pronatrice et une rupture au niveau de la plaque de croissance de la tête ulnaire. Le traitement se base sur la réduction de la fracture sous anesthésie complétée par une immobilisation plâtré. Lorsque la réduction reste impossible une réduction chirurgicale s'impose. La lésion de la physe peut se traduire à long terme par l'installation de trouble de croissance de l'avant bras traumatisé suite à la lésion du cartilage de conjugaison. D'où l'intérêt de la réduction anatomique et la stabilisation urgente de la fracture.

**Figure 1:**
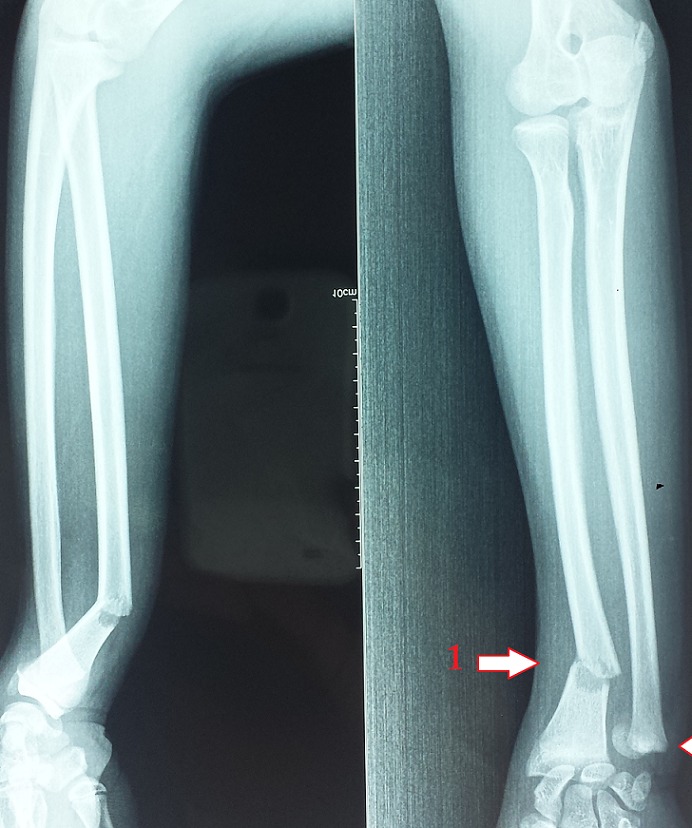
Radiographie de l'avant bras de face et de profil montrant une fracture de la diaphyse radiale avec une angulation antérieure (flèche 1), associée à un décollement épiphysaire stade II de Salter et Harris de l'extrémité distale de l'ulna (flèche 2) soit une fracture équivalente à la fracture de Galeazzi

